# Quick Curing Mechanisms for All-Season Paints and Renders

**DOI:** 10.3390/ma15207397

**Published:** 2022-10-21

**Authors:** Ivan Cabrera, Markus Rückel, Volodymyr Boyko, Roland Baumstark, Immanuel Willerich

**Affiliations:** BASF SE, Carl-Bosch-Straße 38, 67056 Ludwigshafen, Germany

**Keywords:** early rain resistance, waterborne coatings, drying, latex, all-season paint, colloidal stability

## Abstract

Paints and coatings are required to quickly cure under a broad variety of environmental conditions and deliver solid long-term performance. Achieving a balance during all seasons between quick curing of a coating film, i.e., early rain resistance, while maintaining sufficient workability and open time for an optimized aesthetic appearance is a challenge for the architectural coatings industry. This article describes how the colloidal physics differs between the current standard mechanism to achieve early rain resistance by inhibited coagulants in winter paints and a new mechanism that provides all-season paints. A combination of advanced physical characterization methods, such as electrophoretic mobility, dynamic light scattering and confocal laser scanning microscopy, in combination with application tests, is used to provide a comprehensive mechanism of the early rain resistance achieved by such paints. In addition, it is shown that this new system can be transferred to wood coatings and organic renders. The key finding of this article is that all-season paints combining early rain resistance at cold and damp conditions with open time at high temperatures and dry conditions rely on fast paint film formation with high early integrity rather than coagulants triggered by base evaporation.

## 1. Introduction

**Figure 1:**
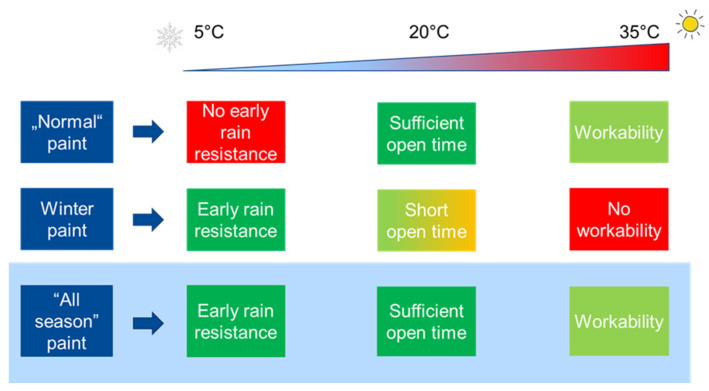
Criteria for all-season paints.

Nowadays, the coatings industry is facing many challenges, first and foremost, becoming climate-neutral by reducing the carbon footprint to net zero and using sustainable raw materials while further improving product performance [1]. Furthermore, due to the intensifying shortage of skilled contractors, the efficiency of coating application processes must be increased and the complexity of construction projects reduced [2]. The above points are valid for all kinds of coatings, e.g., masonry, exterior wood, renders, flexible roof coatings, etc. One way to achieve the labor shortage aspect is to extend the painting season by enabling contractors to work in both cold and humid and warm and dry weather conditions. The key element to reducing the complexity is if paint manufacturers use only one paint formulation throughout the year, avoiding storage management and training of contractors for the specifics of two different winter and summer paint types. Therefore, an all-season paint is needed, as defined by the criteria in Figure 1. In this article, the physical mechanism of winter paints using traditional inhibited coagulants in combination with volatile organic bases is compared to a new mechanism for an all-season paint that does not require volatile bases for storage. To accomplish this, application tests of early rain resistance (ERR) for a masonry façade paint are combined with advanced analytical methods to understand the connection between performance and colloidal physics. Namely, confocal laser scanning microscopy (CLSM), dynamic light scattering (DLS) and electrophoretic mobility are employed to study the colloidal interactions and the drying and rewetting dynamics of façade paint films for masonry applications. Further examples from the fields of wood coatings and renders show that the same mechanism, striking a balance between early rain resistance and open time under critical temperature and humidity conditions, is also applicable for such coatings, without compromising on other performance properties.

### Early Rain Resistance Systems

**Figure 2:**
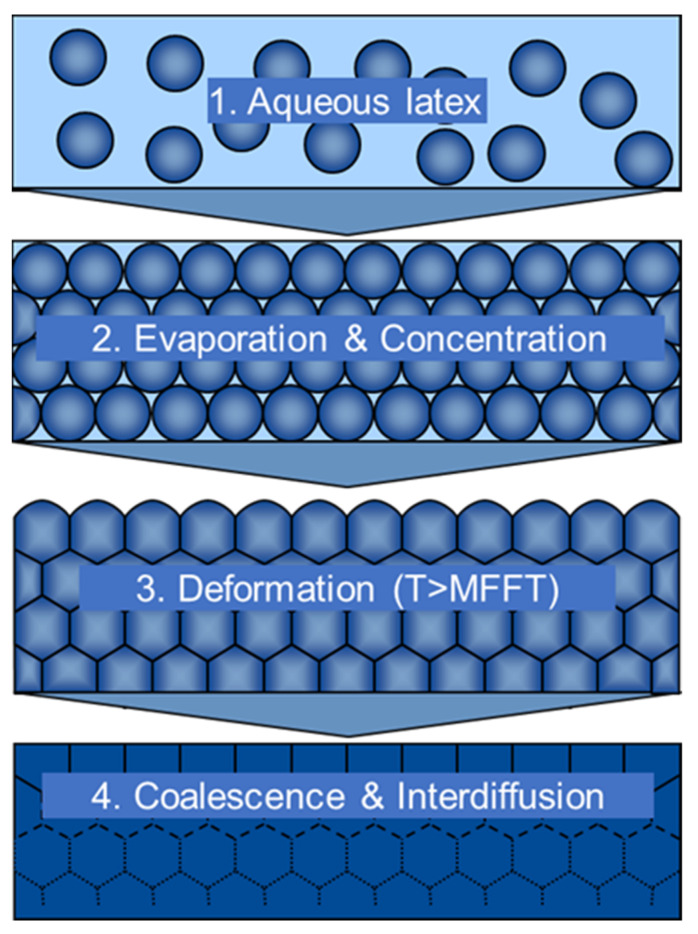
Stages of film formation for latex particles. T is the temperature during the film formation. MFFT is the minimum film formation temperature of the latex.

To understand early rain resistance, the complex process of film formation for polymer lattices is key [3]. Figure 2 schematically depicts the stages of film formation of a latex dispersion. In the first approximation, the same process also applies to waterborne paints and coatings in focus of this work, as waterborne latex serves as a binder for such formulations. Before the film formation in stage 1, the waterborne latex (paint) is in a colloidally stable state and the particles can still switch their positions. This is common at this stage when the paint is freshly applied to the wall. If instantaneous ERR is desired, only 2k systems inducing instantaneous coagulation of the colloidal system are feasible because 1k systems would have otherwise already coagulated in-can during storage. A common example of an instant setting system is the mixing of a coating formulation with a multivalent metal ion solution, e.g., aluminum salts, followed within seconds by a spray application of the paint to the substrate. The underlying curing mechanism in such 2k systems is electrostatic destabilization of the latex by multivalent ions and, in turn, rapid coagulation, leading to the expulsion of the water from the solid paint material [4]. One step further into the film formation process, in stage 2, a portion of the water has already evaporated and the particles start to touch each other. Thus, coagulants inhibited by volatile bases can be used because a part of the volatile base evaporates simultaneously with the water. Such systems are well known and usually use functionalized polyamines, e.g., (ethoxylated) polyethylene imines, polyvinylamines or poly(oxazolidinylethyl methacrylate), which are usually combined with excess ammonia as the volatile base [5,6,7,8]. By using such inhibited coagulants in latex paints, early rain resistance is achieved before or in the early stage of deformation and interdiffusion (step 3). When the pH decreases and the inhibited polyelectrolyte coagulant becomes activated by acquiring a positive charge, the coagulation process of the colloidal particles within the paint formulation starts [9]. Both the above-mentioned 2k systems and inhibited coagulants are effective but not usable under hot and dry conditions as the coagulation effect, which is desired in the winter season, leads to a loss of workability and open time in the summer. The main focus of this work is the detailed mechanistic investigation of a new binder system that enables latex paints to be used in the winter and summer seasons. This binder system relies on the combination of a specially designed latex mixed with a tertiary polyamine [10]. Its mode of action most likely involves stages 3 and 4, shown in Figure 2, namely, the early formation of a robust paint film with resistance against water. This process takes place when the ambient temperature, T, is above the minimum film formation temperature (MFFT).

## 2. Materials and Methods

### 2.1. Materials

For the latex dispersions, all mentioned samples were obtained from BASF SE, Ludwigshafen, Germany. All of the provided particle sizes result from dynamic light scattering, as described in Section 2.2. For the façade paint application, the commercial pure acrylic dispersion Acronal EDGE 6390 was used (binder D; a particle size of 155 nm, a solids content of 50%, BASF SE), which contains a tertiary polyamine, as described in [10]. The comparative experiments were carried out with a standard pure acrylic latex dispersion binder, Acronal A 684, without a tertiary polyamine (binder A; a particle size of 100 nm, a solids content of 50%, BASF SE) and with the base latex of Acronal EDGE 6390 without adding the tertiary polyamine (binder C). The comparative experiment with an inhibited coagulant was carried out with a blend of binder A and an ethoxylated polyamine coagulant, as described in Ref. [5] (=binder B). For the wood coatings, the new Acronal 6391 binder was used (binder F; a particle size of 90 nm, a solids content of 44%, BASF SE), which is an acrylic binder mixed with a tertiary polyamine. The comparative in this case was the acrylic binder Acronal LR 9014 (binder E; a particle size of 80 nm, a solids content of 45%, BASE SE). For the plasters, Acronal 5560 (binder H; a particle size of 166 nm, a solids content of 50%, BASF SE) was used, which is also functionalized with a tertiary polyamine. As a comparison, the standard styrene acrylic Acronal S 790 binder (binder G; a particle size of 150 nm, a solids content of 50%, BASF SE) was chosen. For the further paint components, the paints were formulated with a variety of common market-available pigments, fillers and additives. The formulations of the test paint, wood lacquer and render are provided in Appendix A, along with available information on the chemical composition of the ingredients.

### 2.2. Methods 

**Figure A1:**
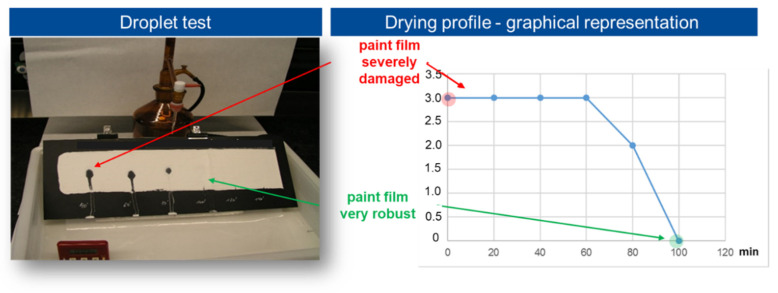
Setup for early rain resistance testing for façade paints.

For the ERR testing of the exterior paints, the paint film was doctor-bladed on a pre-weighted black 8 cm wide Leneta foil (Leneta Company Inc., Mahwah, NJ, USA) with an automated doctor-blading machine using a doctor blade with a 300 µm gap width (both Zehntner GmbH, Sissach, Switzerland). The drawdown length was 30 cm. It was confirmed that the amount of applied coating in weight was comparable to ensure consistent results. The control experiments were conducted within the weight range found for multiple drawdowns (±10% applied weight to the same area) to ensure that such variations in the applied weight do not influence the results. The foil was then left either on a lab bench (25 °C) or in a refrigerator (5 °C) for an initial waiting and drying period and then tested in a droplet test after the defined times. Testing was performed with the same film that was afterwards returned to its drying place (bench or refrigerator) before being tested again. Testing was performed by placing the foil onto a metal support plate, tilting it to an angle of 45°, and then dropping 5 mL of water from a 25 mL burette (VWR International, Radnor, PA, USA) for 1–1.5 min onto the paint. Care was taken to leave enough distance between the edge of the film and the point where the water droplets hit the paint film. The grading was performed from 3 (black Leneta foil exposed), 2 (paint layer partially removed, but black Leneta foil not visible), 1 (only a small change on the paint surface, but some white color of the water running off the Leneta observable as run-off marks) and 0 (no water marks on the paint film and no run-off). Intermediate non-integer grades were given for the intermediate states. The setup is depicted in Figure A1 in Appendix B.

**Figure A2:**
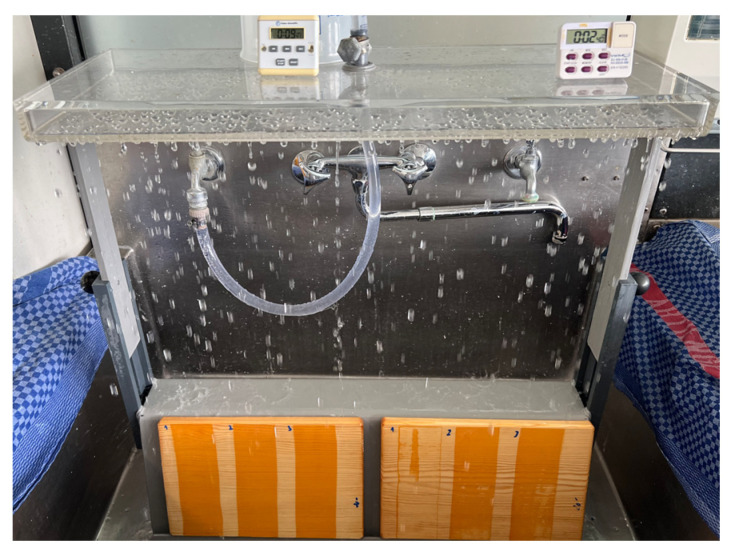
Setup for early rain resistance testing for wood paints.

For the ERR testing of the wood paints, the paint film was doctor-bladed onto wooden panels (pine), conditioned overnight at 5 °C and 75% relative humidity, with a 200 µm doctor blade. After defining the drying times in a Weiss SB 11 climate chamber (Weiss Technik GmbH, Reiskirchen, Germany) at 5 °C and 75% relative humidity, the panels were put into a home-built rain chamber (Figure A2) and photographed after a 5 min exposure to artificial rain and subsequent re-drying.

For the ERR testing of the renders, fiber cement (Eterplan, Etex Germany Exteriors GmbH, Heidelberg, Germany) plates with dimensions of 200 *×* 100 × 5 mm (3 d watered, cleaned and dried for 24 h) were coated with a fine brush rendering. After drying, a conventional façade blue paint was applied for a colored background. The panels were then left to dry for at least 14 days. The fiber cement panel was placed in a climate chamber at the desired testing conditions (e.g., +7 °C and 90% humidity) for 24 h before performing the test. A cooled test panel was taken out of the climatic chamber and placed on a balance. The render to be tested was applied quickly with a spatula (46 g/200 cm^2^), after which it was distributed with a stainless-steel trowel and rubbed up to the thickness of the grain with a plastic float and then quickly placed back in the climate chamber. After the required drying time (e.g., 30 min), the panel was removed from the climate cabinet and placed vertically in a watering system, and 1800 mL of water was run down on the panel for 15 min. The water used for every panel was collected and stirred, and 210 mL was transferred into a glass jar. The procedure was repeated for every render until the collected water was not turbid anymore, and the time was documented.

The drying kinetics were measured by repeatedly weighing the films prepared in the same way as the film for the ERR testing, after defining the drying times on a precision balance (Sartorius GmbH, Göttingen, Germany).

For the confocal laser scanning microscopy (CLSM) studies, the paint formulation was stained with a 200 ppm Nile blue before the coating. For each paint formulation, two coatings on the Leneta foils were prepared; one for the CLSM study and another for the simultaneous drying measurement by gravimetry. After 4 different time points of drying, 30 min, 40 min, 50 min and 70 min, the coated foils were cut into smaller pieces for the subsequent microscopic study. Before the imaging process was started, a small droplet of water was put onto the partially dried paint surface and covered by a thin glass slide. The imaging was performed with an upright microscope (Leica SP5) using a water-immersion objective lens (63×, 1.2 NA), both from Leica GmbH, Wetzlar, Germany. The two imaging contrasts were recorded over time in the xyt mode. One of the contrasts is based on the backscattering of a laser at a wavelength of 458 nm, which allows for the visualization of the distribution of individual TiO_2_ particles, and the other relies on the fluorescence of the Nile blue, excited at 633 nm, to visualize the binder distribution. The fluorescence was collected in the spectral window between 639 nm and 795 nm. In order to increase the frame rate, the scan speed was set to 700 Hz, and the image size was reduced to 40 µm × 20 µm. For the in situ study of the drying kinetics of the pure binders, fluorescently stained polymer dispersions (stained by a 300 ppm fluorescein sodium salt) were coated onto precision cover glasses (thickness No. 1.5H) by using a doctor blade with a 280 µm gap size, and they were directly studied on the microscope stage (Leica SP8, inverted setup; Leica GmbH, Wetzlar, Germany) by using the xzt scan mode. The image analysis to determine the film thickness over the drying process was executed by using Matlab (MathWorks, MA, USA). For each xz image of the xzt series, the upper and lower interfaces of the film were determined by averaging along the *x*-axis, subsequently differentiating the intensity profile along the *z*-axis, as well as localizing the minimum and maximum of this differentiated intensity profile. The difference between the upper and lower interfaces directly yields the thickness of the film.

The dynamic light scattering measurements were carried out using a compact ALV CGS 3 setup equipped with a He–Ne laser with a wavelength of λ = 632.8 nm and with 22 mW output power, ALV electronics and a correlator (ALV GmbH, Langen, Germany). The measurements were taken at an angle of 90°, and the data were evaluated using the cumulant method. The measurements were all carried out at a 0.001% solids content, diluting the pure lattices with deionized water; the pH was adjusted with 100 mmol/L NaOH or HCl solution [11,12].

The electrophoretic mobility measurements were carried out using a Zetasizer Nano (Malvern Instruments GmbH, Herrenberg, Germany). A 4 mW 633 nm He–Ne laser was used, and the detection angle of the outcoming light was 13°. The measurements were all carried out at a 0.1% solids content in 10 mmol KCl solution, and the pH was adjusted with 100 mmol/L NaOH or HCl solution.

For the surface pH measurements, an InLab surface electrode (order number 51343157) and a FiveEasy standard pH measurement device, both from Mettler Toledo, Gießen, Germany, were used to follow the pH of the paint films, prepared as described for the ERR measurements.

## 3. Results and Discussion

### 3.1. ERR and Workability Testing of Paint Samples

**Figure 3:**
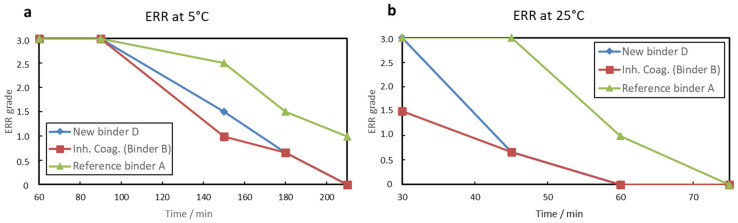
ERR rating of the paints with the reference binder A, the reference binder with inhibited coagulant (Binder B) and the new binder D (containing tertiary polyamine), formulated as shown in Table A1. The tests were carried out according to Section 2.2. (**a**) The humidity at 5 °C was approximately 50%; (**b**) at 25 °C, the humidity was approximately 45%. It should be noted that a small difference in the humidity between 5 and 25 °C was accepted to avoid differences in air movement above the drying films by active ventilation, the latter having a far greater effect on the time to ERR than the former. This is not possible when using a humidity-controlled chamber due to the much lower absolute humidity at 5 °C vs. 25 °C, which requires faster ventilation to hold the relative humidity constant; thus, artificially accelerating the time to ERR at 5 °C.

**Figure 4:**
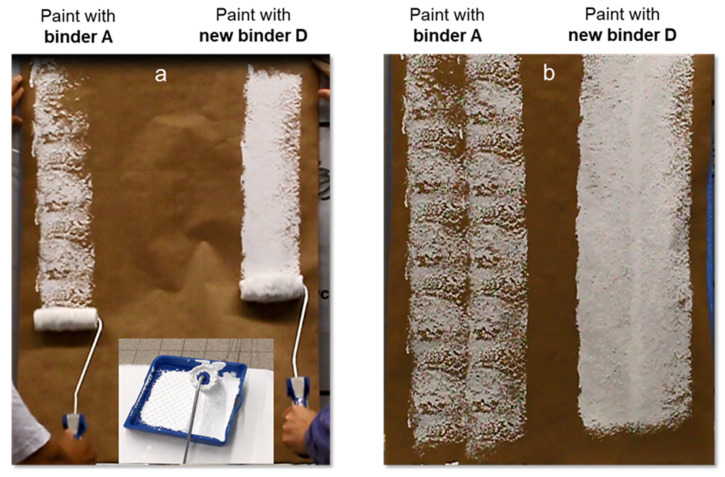
Comparison of the open time of paints with the reference binder A and the new binder D (**b**). The paint rollers were saturated with the same amount of the paint formulation from Appendix A with both binders and rested for 40 min before applying them to a paper substrate (**a**).

As discussed in the introduction, the scope of this work is to compare the early rain resistance of paints produced with the standard acrylic binder A, as well as with binder B, which is binder A equipped with an inhibited coagulant system based on ethoxylated polyethylene imine combined with excess volatile base ammonia, and with the new acrylic binder D equipped with tertiary polyamines. In Figure 3, it is evident that both paints containing either binder B with the inhibited coagulant or the new binder D with the tertiary polyamine become rain resistant earlier than the reference binder A at high and low temperatures. While the binder with the inhibited coagulant (binder B) is slightly faster on the way to early rain resistance, the full ERR (grade = 0) is achieved at the same time as the new binder D. Being much faster at higher temperatures is even undesirable, because at 25 °C and above the open time is important, i.e., an ERR grading of 1.5 after only 30 min indicates a lack of workability. This is already the first indication that traditional ERR-optimized (winter) paints with inhibited coagulants are not feasible as all-season paints. In contrast, the new binder system D combines better open time and workability at high temperatures with similar performance at low temperatures, which is visible in Figure 4. There, the unique application properties of the new binder D concerning workability over paints with binder B can be observed. When both paints are applied at 25 °C, after being loaded as a thin film on a roller and idling for 40 min, the paint with the new binder D is still workable. This means that most of it can still be applied to the substrate, resulting in a white substrate. The paint with the reference binder A is clearly inferior as it is obvious from the lower amount of paint that transfers to the paper substrate due to the lack of workability and open time after idling on the roller. The paint with binder B that contains the inhibited coagulant cannot be applied at all after idling under the same conditions for 40 min on the roller.

### 3.2. Mechanistic Study

To elucidate why the paints produced with the new binder D can offer the advantages of classic winter paints while not having their drawbacks, a combination of modern analytical methods was used. Firstly, the colloidal physics is investigated on the nanoscale using electrophoretic mobility and dynamic light scattering studies. Secondly, CLSM is used to understand the drying process on the micrometer scale.

#### 3.2.1. Colloidal Stability and Electrostatic Interactions

**Figure 5:**
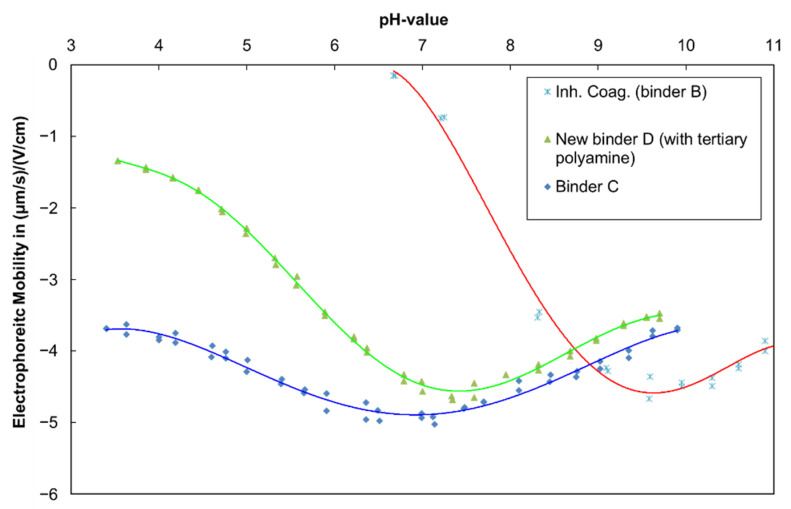
Electrophoretic mobility of the new binder D, binder C (without a tertiary polyamine) and binder B with an inhibited coagulant. The mobility curve for binder A is very similar to binder C.

**Figure 6:**
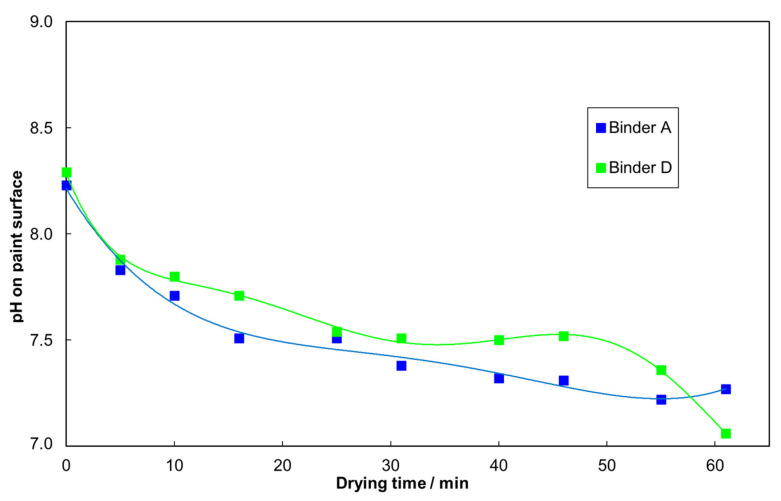
Evolution of pH during drying of the façade paint formulation from Table A1 each with binder D (green) and with the reference binder A (blue). The pH was measured on the coating surface using a surface pH electrode.

First, the electrophoretic mobility is used to study the electrostatic interactions in both of the ERR-enabling binder-additive systems discussed in Section 3.1, i.e., binders B and D. The electrophoretic mobility curve in Figure 5 shows that the inhibited coagulant in binder B leads to a steep change in the electrophoretic mobility, starting between pH 9 and 10. The reason for this is that the acid constant (pKa value) of the primary and secondary amines incorporated into the polymers is in this range [13]. When the pH decreases, an increasing portion of the inhibited coagulant becomes activated by acquiring a positive charge, which decreases the surface charge of the negatively charged latex. This, in turn, reduces the negative electrophoretic mobility, which is correlated to colloidal stability in mainly electrostatically stabilized colloidal systems. Between pH 9 and 7, the electrophoretic mobility of this system approaches zero, indicating diffusion-controlled coagulation in electrostatically stabilized systems without a steric contribution [14]. Therefore, ammonia is needed to keep the system storage stable at a pH above 10, which requires high amounts of this base and, in turn, leads to a strong odor. In contrast, the new binder D containing the tertiary polyamine, which has a lower pKa value [13], does not have a significant effect on the electrophoretic mobility of the latex above a pH of 7. Thus, the mechanism for binder D using tertiary polyamines is different from the inhibited coagulant, and there is no need for a volatile base as a trigger. From Figure 6, it is evident that during drying, the pH trace of the paint with the new binder D does not appear different from binder A, i.e., the effect of the tertiary polyamine on the electrophoretic mobility becomes relevant only in the final stage of the drying process when the pH approaches 7. In summary, at the paint storage pH between 8 and 8.5, the new binder D does not interact with the tertiary polyamine according to the electrophoretic measurements, which is in stark contrast with binder B, where the inhibited coagulant requires a pH of around 10 to prevent paint coagulation during longer storage periods.

Further proof for the lack of interaction between the latex and the tertiary polyamine in binder D is derived from dynamic light scattering, as shown in Table 1. The hydrodynamic diameter of the pure latex (binder C) does not change between pH 8.5 and 6.5. Only when the tertiary polyamine is present, as in binder D, a slight change in the hydrodynamic diameter and the polydispersity occurs, showing the onset of interaction when the pH is changed from 8.5 (storage conditions) to 6.5 (late drying process). Thus, the new binder D system, containing a soft coagulant instead of a hindered one, preserves the advantages of storage stability at standard pH, open time and workability at high temperatures while enabling ERR at low temperatures. It should be noted that the light scattering experiment at low concentrations is only a qualitative result that cannot be extrapolated to high concentrations in the paint that are more than 100 times higher.

#### 3.2.2. Drying Process and Water Sensitivity

While Section 3.2.1 describes in detail how the interactions between the latex and the additive differ between the inhibited coagulants based on ethoxylated polyethylene imines and tertiary polyamines, this is not the only aspect of why both systems behave differently. Otherwise, adding the tertiary polyamine to the reference binder A would have to yield superior ERR, as it does in binder D, which is, however, not the case. In fact, adding the same amount of tertiary polyamine to the reference binder A does not improve the ERR significantly. The reason for this is that the latex in binder D also contributes to the unique performance of the paint produced with the latter.

**Figure 7:**
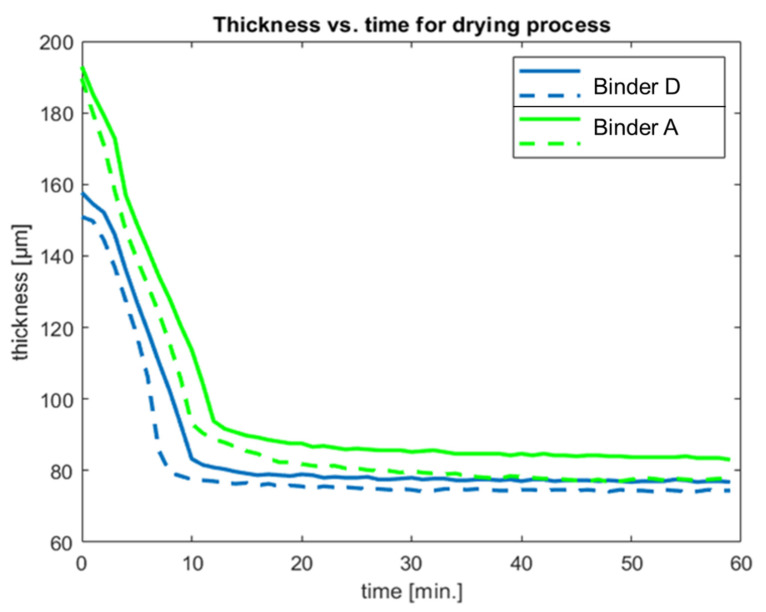
Drying kinetics of the reference latex A and the new binder D. Each binder was studied twice (solid and dashed lines). The thickness decline over time is very similar for all of the curves shown.

**Figure A3:**
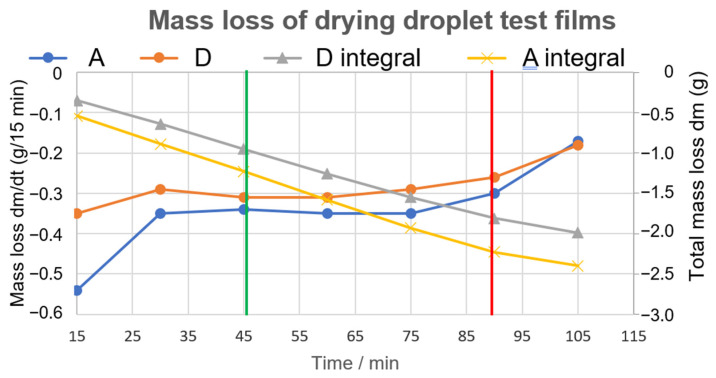
Gravimetric drying kinetics study recorded from films drawn on Leneta foil with a 400 µm doctor blade. The amount of paint applied for binder A was 11.85 g and 11.13 g for binder D. A denotes the paint with reference binder A; D denotes the paint with the new binder containing a tertiary polyamine. The green vertical bar is the latest point in the drying time when the paint behaves as a liquid (from touching with a glove); the red vertical bar is the touch dry point; whereas, in between, the irreversible marks remain on the touched paint surface.

To approach this topic, CLSM is a suitable tool. Figure 7 shows the drying kinetics of the new binder D versus the reference binder A. The films of the reference binder A and binder D were drawn using the same doctor blade. The resulting films were slightly different in thickness for both binders, the reason being minor rheological differences. As this experiment is not performed with the highly light scattering paints but with the pure binder lattices, the turbidity of the drying latex is low enough for the laser light of the CLSM to penetrate the whole film depth. Thereby, it is possible to follow the film drying by the decline in film thickness. When comparing both of the latex drying curves, no significant differences are visible. Thus, it can be ruled out that early coagulation is the reason for the advantage in the ERR of the new binder system. In that case, a faster drying process would be expected as the coagulating solid and the water phase then separate and increase the water evaporation, which is not the case. This conclusion of similar drying kinetics is also confirmed by gravimetric drying experiments, shown in Figure A3 in Appendix B.

Moreover, the complimentary experiment of rewetting the binder films provides important insights. A new fluorescent dye, 1,2-bis[4-(3-sulfonatopropoxyl)phenyl]-1,2-diphenylethene (BSPOTPE), was used that allows to monitor the water uptake of dried latex films. It is beyond the scope of this work to describe this dye in more detail, which will be described elsewhere. The selected dye is strongly fluorescent when it is in a dry environment and almost non-fluorescent when it is in a wet environment. When films, such as the one described in Figure 7—dried for 60 min but stained with this special dye—come into contact with water, the penetration into the dried film can be followed over time by CLSM. The results of such an experiment show that within 80 s after exposure to water, only 30 µm of the film of the new binder D is penetrated by the water, whereas for the film of the reference binder A, 70 µm is penetrated within the same time period. This proves that the early resistance against penetration by liquid water is the key to a superior ERR, i.e., while the reference binder A cannot prevent rapid rewetting and, in turn, partial or complete redissolution. The new binder D forms a film that exhibits high early water resistance despite containing a tertiary polyamine. Thus, early film resistance is key to success with ERR mechanisms based on engineered binders and “soft” coagulants.

**Figure 8:**
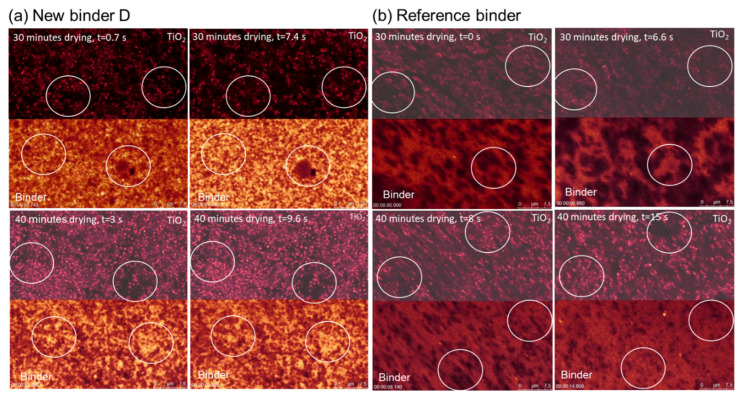
Rewetting kinetics of partially dried paints. If the paint with binder D is followed for a longer exposure time, the result does not change, i.e., no redissolution occurs. Circles mark the areas where constant or changing binder and TiO_2_ particle positions can be observed best.

This can be further elucidated on the microscopic level by observing the surface of the paint. Figure 8 shows the rewetting of a partially dried paint film on its surface after drying for 30 and 40 min. After 30 min of drying, the water droplets are freshly placed onto the paint surface, and the mobility of the main paint components (binder and titanium dioxide (TiO_2_) particles) was imaged by the CLSM setup. The left image for each paint was recorded immediately after the addition of the water droplet; the right one was recorded a few seconds later. It is evident that the TiO_2_ and the binder pattern change with time when comparing the pairs of images in the top row for the new binder D versus binder A, i.e., the particles in the paints show movement in the white circles (the positions were arbitrarily chosen and facilitate better eye guidance), marking the same spot in the image. Their change with time indicates an immediate redissolution of both paints with binders A and D. After 40 min of drying, the addition of the water droplet does neither change the pattern of binder D nor the TiO_2_ distribution in the corresponding white circles in the right image. For the reference paint with binder A, the added water droplet still disrupts the paint film surface after 40 min of drying, and the binder and TiO_2_ particles start moving again, resulting in different arrangements of both species in the white circles in the image on the right. This corresponds to the previous experiment with the pure binder, confirming that water sensitivity is a key element in achieving ERR.

In conclusion, for the mechanistic part of this work, it could be proven that a new binder system based on a tailored latex and a tertiary polyamine “soft” coagulant allows for the formulation of a true all-weather paint, as opposed to earlier systems tailored to cold conditions. The key to ERR at low temperatures and workability and open time at high temperatures lies in the use of a polyamine that becomes activated only in the late stage of drying due to its low pKa value, as compared to traditional coagulants, in combination with a latex that delivers exceptional early film water resistance after short drying times.

### 3.3. Binders and Guiding Formulations for Wood Paints with Outstanding ERR

The base concept to gain early rain resistance by using the same tertiary polyamine functionalization is also of interest for exterior wood coatings. Here, the request to speed up film formation is even more demanding as exterior wood stains normally have a limited solids content of 20 to 35%. Even opaque wood coatings often have a limited solids content of just 40 to 50%. This means that more water must evaporate in general before a setting of the wood coating film is achieved. It also shows that the risk of washing off the freshly applied wood stain or wood paint by an early rain event or water contact is, in general, even more pronounced than for exterior masonry paints of a higher solids content (up to approximately 70%). Most often, a minimum drying time of 1 h is needed to gain some stability against redispersion of the wood coating by water for conventional formulations. Classical solutions to achieve quick early rain resistance by using inhibited coagulants, e.g., by (modified) polyethylene imine, limit, in any case, the open time, as described for exterior paints in Section 3. Furthermore, as a consequence of the fact that to obtain some stability in the viscosity of the final blend, a pH of at least 10 or 11 must be adjusted using a strong load of the evaporable base ammonia. This is, from an odor point of view, often seen as unacceptable for the applicator. The high pH may also lead to damage and discoloration of the wood substrate, which is especially negative for clear coats and semi-transparent lightly colored stains. Moreover, this speeding up of the film formation comes along with a restriction in the open time under normal drying conditions.

**Figure 9:**
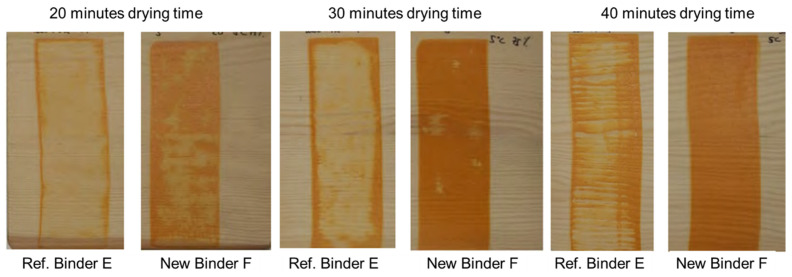
ERR test of wood stains, formulated according to Table A2 and tested according to Section 2.2. The temperature during drying was 5 °C, and the humidity was approximately 75%.

**Figure 10:**
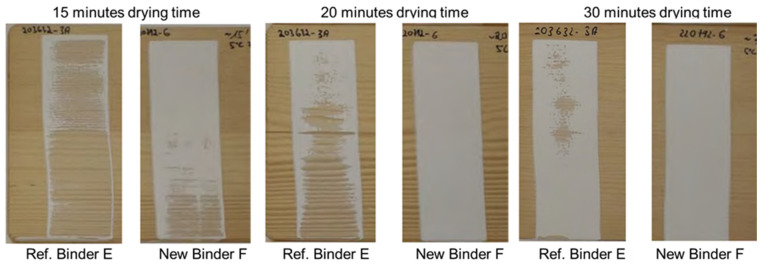
ERR test of white wood paints, formulated according to Table A3 and tested according to Section 2.2. The temperature during drying was 5 °C, and the humidity was approximately 75%.

Starting from a multi-phase acrylic binder concept, which is also the basis for the well-known market product Acronal LR 9014, a new binder was developed using the same tertiary amine functionalization to generate the more weather-tolerant type of binder F, called Acronal 6391. In the first step, an optimization of the reactivity was needed to find some way of speeding up the film formation without sacrificing the workability and the open time. By tailor-making the binder, early rain-resistant robust base formulations for wood stains and white exterior wood paints could be created (see Table A2 and Table A3 in Appendix A). As shown in Figure 9 and Figure 10, the early rain resistance could be improved to an unprecedented level. While keeping the overall performance profile, the rain resistance after the film formation at adverse conditions of 5 °C and 75% relative humidity was much better for the new binder F than for the reference binder E. The optimization of the new binder F came along without suffering significantly in open time at normal summer drying conditions, which is a clear advantage over classic inhibited coagulant technology. To further improve the workability and the open time, even the use of 1% of the solvent propylene glycol is possible without noticeably increasing the time needed to achieve early rain resistance.

### 3.4. Binders and Guiding Formulations for New Renders with Outstanding ERR

**Figure 11:**
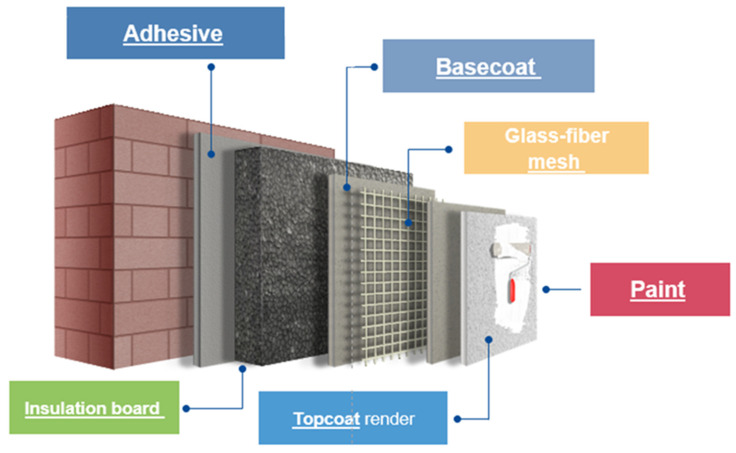
EIFS system with all components. The topic of this section is the topcoat render.

As mentioned in the introduction, one of the many challenges that the coating industry faces is to become climate-neutral. In this respect, helping to minimize the energy demand of buildings is very important. External wall insultation is a way to achieve this, and external thermal insulation composite systems (ETICSs) have been used for many years to construct energy-efficient buildings. The most commonly used ETICSs are applied to solid walls built from masonry or concrete and use a rendering system as the final layer. Nowadays, if organically bonded finishing is used, then it is usually selected in the final color of choice. A final layer of paint may also be used to improve the system, as is the usual case during renovation. In Figure 11, an illustration of a typical system is shown. From a consumer point of view, the development of ERR renders as part of ETICSs can also contribute to increasing the number of energy-efficient buildings by increasing the contractor capacity. In Section 3 of this article, the paint was described; here, we focus on the topcoat render.

In the development of renders with high ERR performance, the differences in thickness and binder content between the paints and renders play a very important role. Renders are generally applied to the thickness of the coarser filler, which can be several millimeters. Paint films are considerably thinner. Thus, render drying usually takes much longer. Independently of these differences, it should be clear that the application of exterior coatings should not be carried out during the real winter season, when temperatures are likely to fall below freezing. The problem under these conditions is that even if some film formation is possible, the render would have to be flexible to such an extent that the strength of the bond between the binder and the fillers is sufficient to be able to absorb the spatial expansion of the water associated with ice formation. If the frost acts at a time when this strength is not yet available, the render structure will be destroyed. In fact, if workability is given, the main problem is not the lower temperature but the frequent alternation between freezing and thawing. Therefore, with all-seasons renders, the aim is not to be able to apply in real winter, but, rather, to extend the working season as much as possible. The challenge is, however, as it was mentioned in the previous part of this article, to offer the right balance between early rain resistance and open time under a wide range of temperature and humidity conditions. Using the technology described for the paints, a new binder, H (Acronal 5560), was developed at BASF for this purpose. Using the formulation given in Table A4, white renders were prepared and tested according to the description in Section 2. The standard styrene acrylic binder G served as a reference.

**Figure 12:**
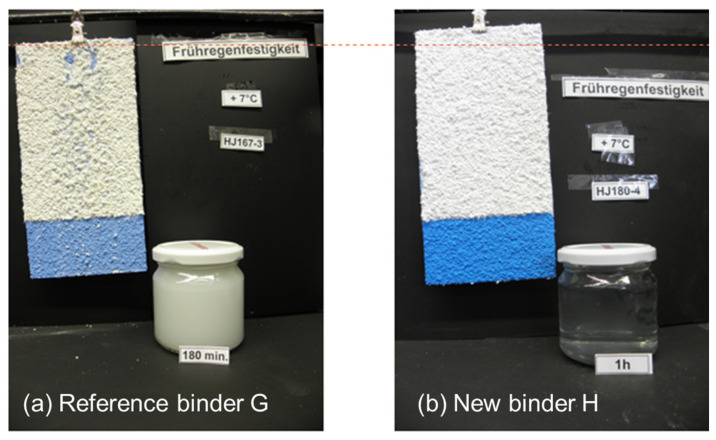
ERR tests of white render topcoats, formulated according to Table A4 in Appendix A and applied and tested according to Section 2.2. Red line marks the highest spot with water exposure.

In Figure 12, the results of the early rain resistance at +7 °C and 90% humidity are shown as an example. As it can be seen, the water collected after watering the render with the new binder H containing tertiary polyamines is clear after 60 min and no surface damage to the white render is seen. In the case of the renders with the standard binders G, even after 360 min, the collected water is still turbid and surface damage to the render is observed. Similar results were obtained at different conditions, e.g., +5 °C and 85% humidity. This clearly demonstrates that the binder technology described here can also be used for renders, with an excellent balance between early rain resistance and workability.

## 4. Conclusions

In this work, it is shown that all-season paints can be formulated using a new type of binder with enhanced early film formation capability in combination with a new tertiary polyamine additive, the latter having a lower pKa value than classical inhibited coagulants, which must rely on volatile base evaporation due to their higher pKa value. An analysis of this mechanism by electrophoretic mobility showed that the new tertiary polyamine in binder D has a pKa value of around 2.5 units lower than that for classic inhibited coagulants, as used in binder B. The dynamic light scattering confirmed this by the fact that changes in the latex particle size for binder D can only be found at a pH of 6.5, while it is stable at a pH of 8.5. This explains why storage-stable coating systems based on the new binders D, F and H can be obtained at a pH of 8–8.5 instead of at a pH of > 10, which is needed for winter paints based on inhibited coagulants. This could be achieved and, especially, without an excess of a volatile base. The mechanism could, thus, be shown to rely on electrostatic interactions that can be triggered at a lower pH than that for standard winter paints. The early rain resistance tests for paints with the new binder D were on par with the paints based on binder B, using technology for long-established winter paints, and significantly better than the standard binder A. In addition, the workability of a paint with the new binder D after pre-drying on a roller at a high temperature was even superior to that with the standard binder A. In contrast, a paint with binder B, which uses inhibited coagulants, solidified in the same test, indicating no workability at all. Furthermore, the CLSM could show that, while water evaporation proceeds at equal speed, binder D also contributes to the better early rain resistance through its higher resistance against water penetration when exposed to it shortly after film formation. In summary, this combination of high and low temperature properties provides access to an all-season paint, relieving the need for winter and summer quality. The described concept enables paint formulators to enhance ERR to a new level in various coating systems, namely, façade paints, wood coatings and render systems. This delivers a contribution to enhanced efficiency and sustainability by preventing environmental pollution by redissolved coating formulations leaking into the ground.

## Figures and Tables

**Table 1:** Light scattering results for the new binder D and binder C without the tertiary polyamine.

pH (T = 25 °C)	Binder	Hydrodynamic Diameter/nm	Polydispersity Index ^1^
8.5	Binder C	156	0.017
8.5	Binder D	156	0.022
6.5	Binder C	156	0.020
6.5	Binder D	162	0.035

^1^ Calculated from the second cumulant fit analysis.

## Data Availability

The authors confirm that all measured data are available within the article. Industrial products where no compositional information is available can be purchased at the respective suppliers.

## Appendix A. Coating Formulations

**Table A1:** Façade paint formulation for binders A to D. The solids content was 68%; the PVC was 55%; the pH was adjusted to 8.2–8.5 using ammonia (and to pH 10 in the case of D2). All brand names are each respective trademarks of the following companies: Shin-Etsu Chemical Co., Inc. (Tylose^®^) (Tokyo, Japan), Kronos International Inc. (Kronos^®^) (Dallas, TX, USA), Omya AG (Omyacarb^®^) (Auftlingen, Switzerland), Imerys S.A. (Paris, France) (Luzenac^®^), Eastman Chemical Inc. (Texanol^®^) (Kingsport, TN, USA) and BASF SE (Acronal^®^, Dispex^®^, Foamaster^®^, Rheovis^®^) (Ludwigshafen, Germany). Distilled water was used (BASF SE, Ludwigshafen, Germany).

Ingredient (Solids) ^1^	Description	Quantity/g
Tylose^®^ MH6000 YP4 solution (0.4%)	Cellulosic thickener	127
Foamaster^®^ MO 2134	Mineral oil defoamer	4
Dispex^®^ AA4040 (40%)	Dispersant	3.5
Kronos^®^ 2190	Titanium dioxide	185
Omyacarb^®^ 2 and 10 GU (1:5)	Calcium carbonate	290
Luzenac^®^ 0	Talcum	55
Acronal^®^ EDGE 6390	Binder	300
Texanol^®^	Coalescent	25
Rheovis^®^ PU1214	Associative thickener	1.0
Water	Medium	9.5

^1^ If different from 100%.

**Table A2:** White wood paint formulation for binders E to F. The solids content was 48%; the PVC was 23%; the pH was adjusted to 8.2–8.5 using ammonia. Additional brand names to Table A1 are each respective trademarks of Evonik SE (Tego Foamex^®^) (Essen, Germany), Eastman Chemical Inc. and BASF SE (ASP^®^, Loxanol^®^). Ammonia and butyl diglycol were obtained from BASF SE, Ludwigshafen, Germany.

Ingredient (Solids) ^1^	Description	Quantity/g
Water	Medium	145
Ammonia (25%)	Base	2
Tego Foamex^®^ 810	Defoamer	4
Dispex^®^ CX4231	Dispersant	10
Rheovis^®^ HS 1332	Acrylic thickener	6
Rheovis^®^ PE 1330	Associative thickener	8.5
Butyl diglycol	Coalescent	10
Loxanol^®^ CA 5308	Coalescent	20
Kronos^®^ 2190	Pigment	130
ASP^®^ G 90	Filler	30
Omyacarb^®^ Extra GU	Filler	60
Acronal^®^ EDGE 6391	Binder latex	562.5
Tego Foamex^®^ 845	Defoamer	3
Ammonia (25%)	Base	0.5
Rheovis^®^ PE 1330 (30%)	Associative thickener	4
Water	Medium	4.5

^1^ If different from 100%.

**Table A3:** Wood stain formulation for binders E to F. The solids content was 31.5%; the pH was adjusted to 8.2–8.5 using ammonia (and to pH 10 in the case of D2). All brand names are each respective trademarks of Kronos International Inc., Sun Chemical Inc. (Luconyl^®^) (Parsippany-Troy Hills, NJ, USA) and BASF SE (Hydropalat^®^, FoamStar^®^, Solvenon^®^).

Ingredient (Solids) ^1^	Description	Quantity/g
Water	Medium	190
Hydropalat^®^ WE 3221	Wetting agent	1
FoamStar^®^ SI 2210	Defoamer	2
Solvenon^®^ DPM	Coalescent	10
Acronal^®^ EDGE 6391	Binder latex	678
Water	Medium	50
Rheovis^®^ PU 1291	Associative thickener	9
Luconyl^®^ NG Yellow 1916	Colorant	27.9
Luconyl^®^ NG Red 2817	Colorant	2.1
FoamStar^®^ SI 2210	Defoamer	1
Water	Medium	29

^1^ If different from 100%.

These are just two examples. Exterior wood coatings of different solids and pigmentation grades from low build to high build and from transparent, via semi-transparent, until opaque are possible with improved early rain resistance by using this new binder technology concept. The pigment volume concentration, PVC, may thereby vary from 0% to 40% at a final solids content from 15 to 55%. Formulation concepts and more information on formulation compounds in use for water-based exterior wood coatings are described in general previously in the literature [15,16,17]. Thereby, all kind of viscosities and rheology profiles are possible depending on the rheology modifier in use. The basic concept used to speed up early rain resistance of exterior wood coatings is not restricted to Acronal 6391. It can also be transferred to other acrylic, acrylic-alkyd or acrylic-PU hybrid exterior wood and trim binders of different elasticity profiles and outdoor durability.

**Table A4:** Render formulation for binders G and H. The solids content was 82.2%; the pH was adjusted to 8.2–8.5 using ammonia (and to pH 10 in the case of D2). All brand names are each respective trademarks of the following companies: Eastman Chemical Inc. (Optifilm Enhancer^®^), Alpha Calcit Füllstoff GmbH (Calcilit^®^) (Köln, Germany), Evonik SE (Tego Phobe^®^) and Thor GmbH (Acticide^®^) (Speyer, Germany). The 4.5 mm glass fiber (wet) was obtained from Schwarzwälder Textil-Werke, Schenkenzell, Germany. The renders can further be formulated with Acronal 5560 and also according to the background given on the renders in Refs. [18,19].

Ingredient (Solids) ^1^	Description	Quantity/g
Acronal^®^ 5560	Binder latex	145
Foamaster^®^ MO 2134	Mineral oil defoamer	4
Tylose^®^ MH6000 YP4 (4% solution)	Cellulosic thickener	31
Dispex^®^ Ultra FA 4430	Dispersant	3
Optifilm Enhancer^®^ 300	Coalescent	10
Butyl diglycol	Coalescent	10
Loxanol^®^ MI 6840	Hydrophobizing agent	5
Kronos^®^ 2044	Titanium dioxide	20
Omyacarb^®^ 40 and 130 GU (1:1)	Filler	390
Tego Phobe^®^ 1500N (50%)	Hydrophobizing agent	50
Glass fibre (wet) 4.5 mm	Fibre	3
Calcilit^®^ 1.0–1.5 mm	Filler	327
Acticide^®^ MBS	Biocide	2

^1^ If different from 100%.
